# Complete genome sequence of *Bacillus thuringiensis* BR145, a strain with insecticidal activity against Lepidoptera pests

**DOI:** 10.1590/1678-4685-GMB-2021-0289

**Published:** 2022-03-16

**Authors:** Ana Paula Scaramal Ricietto, Kátia Cristiane Brumatti Gonçalves, Renan José Casarotto Appel, Daniel Ricardo Sosa-Gómez, Gislayne Trindade Vilas-Bôas, Laurival Antonio Vilas-Boas

**Affiliations:** 1 Universidade Norte do Paraná, Londrina, PR, Brazil.; 2 Universidade Estadual de Londrina, Departamento de Biologia Geral, Londrina, PR, Brazil.; 3 Empresa Brasileira de Pesquisa Agropecuária (Embrapa Soja), Centro Nacional de Pesquisa de Soja, Londrina, PR, Brazil.

**Keywords:** Bacillus thuringiensis, insecticidal genes, virulence factors, Helicoverpa armigera, Chrysodeixis includens

## Abstract

*Bacillus thuringiensis* BR145 isolated from a soybean field in Southern Brazil showed toxicity against two important insect pests from soybean crop, *Helicoverpa armigera,* and *Chrysodeixis includens,* with LC_50_ 0.294 µg.cm^-2^ and 0.277 µg.cm^-2^, respectively. We analyzed the genome of this strain through sequences obtained by Next Generation DNA Sequencing and *de novo* assembly. The analysis of the genome revealed insecticidal genes *cry1Aa, cry1Ab, cry1Ac, cry1Ia, cry2Ab, cyt1,* and *vip3Aa*, suggesting the use of this strain in new strategies of biological control.

*Bacillus thuringiensis* is a Gram-positive bacterium with entomopathogenic activity associated with Cry, Cyt, and Vip proteins, synthesized in the sporulation phase and during vegetative growth. Besides these toxins, *B. thuringiensis* produces virulence factors, which potentiate their pathogenicity, including phospholipases, metalloproteases, hemolysins, enterotoxins, cytotoxins, and others factors (Vilas-Bôas et al., 2007; Palma et al., 2014). Several toxins produced by *B. thuringiensis* strains were described with toxicity to insect larvae of Lepidoptera, Coleoptera, Diptera, and against species of other phyla (Vilas-Bôas *et al*., 2012) and recently the classification of these toxins was revised (Crickmore et al., 2020). Therefore, *B. thuringiensis*-based biopesticides have been used as alternative insect pest control and represent about 98% of formulated sprayable bacterial microbial pesticides (Lacey et al., 2015).

Brazil has emerged as the largest producer and exporter of soybean. Therefore, key soybean pests such as soybean looper *Chrysodeixis includens* Walker, 1858 (Lepidoptera: Noctuidae), one of the most important soybean pests in Brazil (Yano et al., 2016), have a profound impact on insecticide use, as well as the polyphagous pest *Helicoverpa armigera* Hübner, 1805 (Lepidoptera: Noctuidae), which eventually can reach pest status and cause damage, also in cotton and corn (Bueno and Sosa-Gómez, 2014; Pomari-Fernandes et al., 2015). Since the use of safe and more selective insecticides is increasing in the world and is an important demand of the public and farmers, alternative methods of control of these insect pests must be developed. For these reasons, in this study, we performed a characterization of *B. thuringiensis* BR145, a novel strain with toxicity against Lepidoptera pests. 

This strain was isolated in a Brazilian soybean field (Ricieto et al., 2013) and showed entomopathogenic activity in assays with larvae of *Ecdytolopha aurantiana* Lima, 1927 (Lepidoptera: Tortricidae) (Zorzetti et al., 2017a) and *Elasmopalpus lignosellus* Zeller, 1848 (Lepidoptera: Pyralidae) (Zorzetti *et al*., 2017b). Bioassays were performed using lyophilized spores and crystal suspensions against larvae of *H. armigera* and soybean looper *C. includens*. Dilutions of lyophilized *B. thuringiensis* were applied uniformly to the diet surface and allowed to dry. Surface treatments provide doses ranging from 0.02 to 1.05 µg/cm^2^. One neonate larva was placed on the treated surface in each cell of a bioassay tray (128 cells). The trays were sealed with self-adhesive plastic sheets (BIO-CV-16;CD International Inc., Pitman, NJ) and held for at 25 ±1.5 °C. Mortality data were obtained after seven days of exposure. Lethal doses and parameters associated were calculated with Polo software (LeOra Software, 1987).

The strain showed insecticidal activity against *H. armigera* (LC50 of 0.294 µg.cm-2) and to *C. includens* from both origins, with LC_50_ 0.277 µg.cm^-2^, and 0.398 µg.cm^-2^, respectively (Table 1). This LC_50_ of in both species is comparable to previous bioactive *B. thuringiensis* isolates with potential use in microbial control (Ignoffo et al., 1977; Pinheiro and Valicente, 2021).

**Table 1 -:** Concentration/mortality responses of neonate larva to lyophilized *Bacillus thuringiensis* BR145 applied on artificial diet.

Insect	Location	n	Slope ± (SE)	LC50 µg.cm2 (FL 0.05)	LC50 µg.cm2 (FL 0.05)	X2
*H. armigera*	Londrina, PR	224	2.171±0.409	0,294 (0,187- 0,402)	3.470 (1.890-12.088)	0.524
*C. includens*	Araguari, MG	192	2.362±0.478	0.368 (0.280-0.479)	3.557 (1.820-16.084)	3.833
*C. includens*	Campo Verde, MT	192	1.856±	0.277 (0.181-0.452)	4.967 (1.741-106.214)	1.522

^a^ n =number of insects tested.

^b^ Lethal concentration (LC); fiducial limits (FL) in µg.cm^2^ of diet.

^c^ SE= standard error.

Genomic DNA was isolated from strain BR145 using Wizard® Genomic DNA Purification kit (Promega, Madison, Wisconsin, USA) following the manufacturer’s instructions and the DNA library was prepared with Illumina DNA Prep. Whole-genome sequencing was performed by Illumina Hiseq sequencing and the paired-end sequence strategy was chosen, which generated a total of 3,042,174 reads of high quality. The analysis methods were performed according to Zorzetti *et al*. (2015). The genome was assembled *de novo* with SPAdes version 3.9.0 (Gurevich et al., 2013). The final draft genome consisted of 235 contigs (length>1000 bp), with a total size of 6,350,733 bp, N50 value of 84,578, and a G+C content of 34.78%. The RAST server program (Aziz et al., 2008) proposed that this strain contains 6,647 coding sequences and 224 RNA genes in 494 subsystems (Figure 1).

**Figure 1 -:**
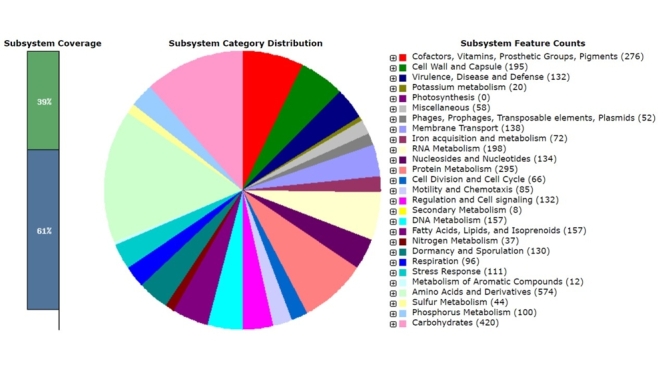
Subsystem coverage and category described by RAST server program in the genome sequence of *B. thuringiensis* BR145.

Sequences that indicate insecticidal genes were identified using Blast tools. Five cry genes were found: *cry1Aa*, *cry1Ab*, *cry1Ac*, *cry1Ia*, and *cry2Ab*, as well as *cyt1* and *vip3Aa* genes. All cry genes and the *vip3Aa* gene were found in plasmid sequences. Genes associated with virulence factirs, such as phospholipases, hemolysins, metalloproteases, and enterotoxins were also located in this genome. The data can be found in genome annotation. Complete genome sequences of several *B. thuringiensis* strains are available on the NCBI Genome website (https://www.ncbi.nlm.nih.gov/genome/genomes/486/). Comparative analysis using BR145 contigs against the nonredundant database identified *B. thuringiensis* serovar *kurstaki* as the closest relative. The complete genome sequence of *B. thuringiensis* BR145 strain has been deposited at GenBank and is available on the NCBI website (https://www.ncbi.nlm.nih.gov/nuccore?term=NZ_PDVK01000001:NZ_PDVK01000235[PACC])

The analysis of the genome sequence and the bioassay results allowed the characterization of *B. thuringiensis* BR145 as a new alternative to be used against a wide range of lepidopteran pests with economic importance, including *H. armigera* and *C. includens*, two important pests causing damages in soybean culture in Brazil.
